# Recurrent Falls in Parkinson's Disease: A Systematic Review

**DOI:** 10.1155/2013/906274

**Published:** 2013-03-05

**Authors:** Natalie E. Allen, Allison K. Schwarzel, Colleen G. Canning

**Affiliations:** Clinical and Rehabilitation Sciences Research Group, Faculty of Health Sciences, The University of Sydney, P.O. Box 170, Lidcombe, NSW 1825, Australia

## Abstract

Most people with Parkinson's disease (PD) fall and many experience recurrent falls. The aim of this review was to examine the scope of recurrent falls and to identify factors associated with recurrent fallers. A database search for journal articles which reported prospectively collected information concerning recurrent falls in people with PD identified 22 studies. In these studies, 60.5% (range 35 to 90%) of participants reported at least one fall, with 39% (range 18 to 65%) reporting recurrent falls. Recurrent fallers reported an average of 4.7 to 67.6 falls per person per year (overall average 20.8 falls). Factors associated with recurrent falls include: a positive fall history, increased disease severity and duration, increased motor impairment, treatment with dopamine agonists, increased levodopa dosage, cognitive impairment, fear of falling, freezing of gait, impaired mobility and reduced physical activity. The wide range in the frequency of recurrent falls experienced by people with PD suggests that it would be beneficial to classify recurrent fallers into sub-groups based on fall frequency. Given that there are several factors particularly associated with recurrent falls, fall management and prevention strategies specifically targeting recurrent fallers require urgent evaluation in order to inform clinical practice.

## 1. Introduction

Falls are a debilitating and costly problem for many people with Parkinson's disease (PD), with people with PD twice as likely to fall as people with other neurological conditions [1]. The consequences of these falls are significant and far reaching, often resulting in injury [2, 3] and contributing to fear of falling [4], reduced activity levels [2], poor quality of life [2, 5], and care giver stress [6, 7]. Given that the prevalence of PD in developed countries is expected to double from 2005 to 2030 [8], PD-related falls can be expected to have a major impact on health care systems in the coming decades.

While it is well known that recurrent falls are a problem for people with PD, the extent and severity of this problem are not well understood. In the general older population, recurrent falls are said to have occurred when an individual falls more than once in a given time period (usually 12 months). Using this definition, around 15% of people in the general older population are classified as recurrent fallers [9]. However, recurrent falls are frequent amongst people with PD, with one study reporting that over 50% of participants fell recurrently [10]. Furthermore, in a survey of 100 people with PD, 13% reported falling more than once per week, with most of these people falling multiple times a day [11]. This suggests that factors underlying recurrent falls in people with PD are different from those underlying recurrent falls in the general population. Consequently, it may be that methods of assessment and classification of fallers, along with fall prevention interventions implemented in the general population, may not be sufficient or appropriate for people with PD.

There are several risk factors known to be associated with falls in people with PD. These include a history of falls, postural instability, freezing of gait, leg muscle weakness, and cognitive impairment [10, 12–16]. However, there appears to be a wide range in the frequency of falls amongst people with PD [17], and there is some evidence to suggest that the risk factors for single falls may differ from the risk factors for recurrent falls [18].

Despite the fact that recurrent falls are a substantial problem for people with PD, the scope of, and risk factors for, recurrent falls in PD are not clearly understood. Previous reviews of falls in people with PD have addressed the overall scope of and risk factors for falls [12, 19]. However, improving the understanding of recurrent falls specifically is the first step towards developing effective interventions designed to reduce and manage these falls. Therefore, this paper aimed to examine studies reporting recurrent falls in people with PD to determine the following.

1. How are recurrent falls classified?
2. What are the rates of recurrent falls?
3. What specific factors are associated with recurrent falls?

## 2. Method

A search was conducted on the 6th and 7th of September 2011 utilizing MEDLINE, EMBASE, CINAHL, AMED, and PsycINFO from the time of their earliest records. The following search terms were used: “PD,” “recurrent falls,” “fall*,” “fall risk,” “repeated falls,” “multiple falls,” and “frequent falls.” Studies included were published journal articles of descriptive or intervention studies including at least 15 participants with PD, and reporting information concerning recurrent falls which was collected prospectively. Studies were considered to have collected falls data prospectively if the data pertained to falls that occurred after the participants entered the study, regardless of the method of falls data collection. Study eligibility was determined in a two-stage process, conducted by one investigator (AKS). Firstly, all study titles and abstracts were screened and studies that clearly did not meet the inclusion criteria were excluded. Secondly, the full article was obtained for the remaining studies and each study was assessed for eligibility. If the investigator was unsure if a study was eligible, a second investigator was consulted (NEA or CGC).

Recurrent falls were defined as having occurred when participants reported more than one fall within the reporting period. Where sufficient data were reported, the number of falls sustained by recurrent fallers (as a group) and the average number of falls per individual recurrent faller were calculated for each study. The reporting period was then used to adjust the data to calculate the number of falls per faller per year (i.e., number of falls divided by the number of fallers, adjusted when necessary to reflect a 12-month reporting period) and falls per participant per year (number of falls divided by the number of participants in the study, adjusted when necessary to reflect a 12-month reporting period). It is acknowledged that this method of adjustment is not ideal as it does not account for the effect of disease progression; however, it facilitates comparison of studies with different reporting periods.

## 3. Results

**Figure 1:**
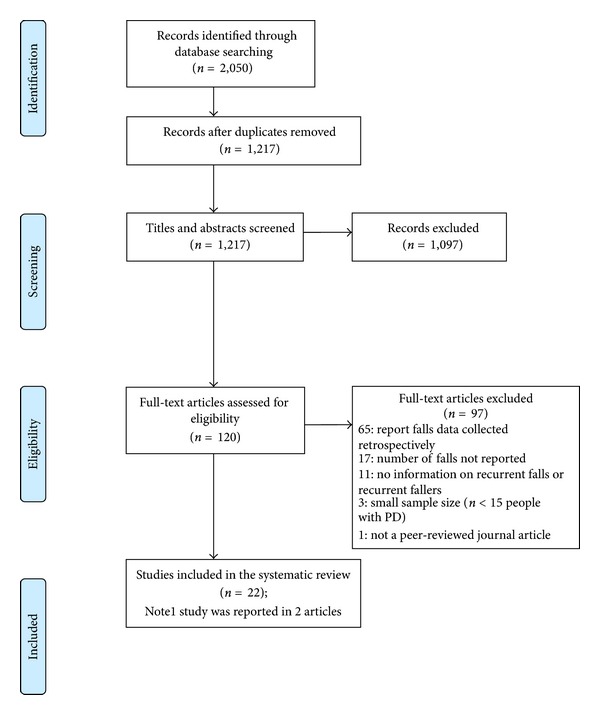
PRISMA flow diagram [38] showing flow of information through the review.

The literature search yielded 1217 results, with 22 studies (Table 1) [2, 10, 13, 15, 16, 18, 20–36] containing information relevant to the review questions (Figure 1 [38]). Seven of the included studies provided information regarding factors associated with recurrent falls (Table 2) [2, 15, 18, 23, 28, 33, 34].

### 3.1. Classifying Falls

Most authors have used aspects of the definition for falls proposed by the Kellogg International Work Group on the prevention of falls by the elderly [37] (Table 1). This definition consists of three components: that a fall is an unintentional or unexpected event, it results in the person coming to rest on the ground or another lower level, and that it is not the result of a major intrinsic event (such as a loss of consciousness) or overwhelming external force. Seven (32%) of the included studies used a definition which incorporated all three components [10, 16, 21, 22, 24, 25, 32, 36], while 3 (14%) of the studies did not provide a definition for falls [23, 27, 31]. 

Sixteen (73%) of the studies classified fallers into groups which separated out participants who fell more than once in the recording period (i.e., recurrent fallers) from participants who did not fall, or who fell once (Table 1) [2, 10, 15, 16, 18, 21–23, 25, 27, 28, 30–35]. Three studies further classified the recurrent fallers into subgroups [30–32]. The time periods over which falls were reported was variable (Table 1); however the most common reporting time was 12 months (11 studies, 50%) [10, 13, 15, 18, 20, 23, 25, 27, 31, 33, 35]. There was one study that recorded falls for 24 months [34] and one that reported from entry into the study until death, ranging from 6 to 29 months [32].

### 3.2. Rates of Falls and Recurrent Falls in Parkinson's Disease

Fourteen (64%) of the studies recorded fall rates using the gold-standard method of a falls diary, calendar or postcard [2, 10, 13, 15, 16, 20–22, 24–26, 29, 30, 35, 36]. Several studies recorded falls via conducting telephone interviews at set intervals, ranging from monthly [18, 33] to three monthly [28, 34] or six monthly [23] intervals. Other studies utilized responses to regular mail queries [27], monthly outpatient follow-up sessions [31], or medical record observation [32].

The proportion of participants who fell at least once during the reporting periods was highly variable (Table 1), ranging from 35% [18] to 90% [20], with an average of 60.5%. Recurrent fallers accounted for between 18% [18] and 65% [31] of participants (average 39%) and made up a large proportion of the fallers, ranging from 50% [2, 16] up to 86% [35] of fallers (average 68%).

The rate of falls per recurrent faller per year was found to be high, ranging from 4.7 [23] to 67.6 [35] falls per recurrent faller per year (average 20.8) (Table 1). An example of the very high rate of falls experienced by some individuals is reported by Goodwin et al. [29], where one participant fell 577 times in 20 weeks, which is approximately equivalent to 1500 falls in 1 year.

### 3.3. Factors Associated with Recurrent Falls in Parkinson's Disease

Seven studies were identified which examined potential factors associated with recurrent falls (Table 2). Six of these studies reported univariate and/or multivariable regression analyses [2, 15, 23, 28, 33, 34], with four studies aiming to identify models which can be used to predict future recurrent fallers [2, 28, 33, 34]. One study [15] aimed primarily to identify variables explaining fall frequency in fallers with PD.

Examination of these studies revealed that a history of a previous fall or falls was a significant factor associated with recurrent falls in all six of the studies that included it in their analysis [2, 15, 18, 23, 33, 34]. It was also found to be a predictor of future recurrent fallers in three of the four studies which aimed to predict recurrent fallers [2, 33, 34].

Disease severity as measured by Hoehn and Yahr stage [39] or by the Unified Parkinson's Disease Rating Scale (UPDRS) [40] was found to be significantly associated with recurrent falls in five of the seven studies [2, 15, 23, 33, 34]. It also predicted future recurrent fallers in half of the studies which aimed to identify predictive variables [2, 34]. One study found that the relative risk of recurrent falls was 13.4 (95% CI 0.4 to 27) for people with Hoehn and Yahr stage 1 to 2.5 and was greater than 100 (95% CI 3.1 to 585) for people at stage 3 to 4 [2]. 

Fall frequency has been analyzed as a continuum in relation to disease severity, age, medications, cognitive variables, orthostatic hypotension, and visual impairment using negative binomial regression in one study [15]. Results showed that disease severity (as measured by the UPDRS), treatment with dopamine agonists, and impaired attention were associated with fall frequency, with associations remaining after adjustment for disease severity. A further study showed an association between fall frequency and cognitive impairment as measured by a Clinical Dementia Scale rated by caregivers [23].

Fear of falling was examined in five of the seven studies [2, 18, 23, 33, 34] and was a significant variable in two of these studies [18, 33]. Fear of falling was shown to be increased in recurrent fallers as compared to single fallers [18], and it was found to be a strong independent predictor of future recurrent fallers utilizing the Activities-Specific Balance Confidence Scale [33]. A cut-off score of 69 on this scale correctly identified 93% of recurrent fallers (sensitivity) and 67% of nonrecurrent fallers (specificity).

Reduced mobility in recurrent fallers was a common theme emerging from between group comparisons. Compared to single and nonfallers, recurrent fallers demonstrated poorer performance on the Functional Gait Assessment [28], the Timed Up and Go [28, 34], and walking speed measures [34]. Recurrent fallers had increased use of walking aids as compared to a group of single fallers [18] and combined single and nonfallers [34]. Additionally, 31% of falls amongst recurrent fallers occurred when using a walking aid [18]. Recurrent fallers also demonstrated reduced walking capacity in terms of six minute walk distance and had reduced speed of standing up from sitting, compared to single fallers [18].

Increased motor impairment as measured by the UPDRS motor score [40] was found to be a predictor of future recurrent falls [33], and recurrent fallers were shown to have increased motor impairment as compared to single fallers [18]. In particular, freezing of gait as measured by the UPDRS item 14 was associated with increased risk of recurrent falls [23]. Reduced physical activity levels, longer disease duration, and higher doses of levodopa have also been observed in recurrent fallers [34].

## 4. Discussion

Recurrent falls are a common problem in people with PD affecting around 70% of people with PD who fall (Table 1). However, there is substantial variability in the falling rates reported in the studies included in this paper, with the proportion of fallers (single and recurrent) ranging from 35 to 95%. This high variability in reported falling rates may be attributable in part to the specific inclusion criteria used in different studies. The study with the highest proportion of fallers included only participants who had PD with dementia [20]. The study with the next highest portion of fallers (86%) included only participants who had experienced more than one fall in the past year, meaning that retrospectively the entire sample was recurrent fallers [29].

Differences in the method of monitoring falls could also contribute to the variability seen in fall rates across the included studies. The falls diary is the preferred method of falls monitoring [9] as it enables falls to be recorded immediately after they have occurred, minimizing the chance of participants forgetting to report a fall. Only 14 (64%) of the included studies used a falls diary or similar monitoring system (e.g., postcards or calendars) [2, 10, 13, 15, 16, 20–22, 24–26, 29, 30, 35, 36]. Several other studies used methods, such as telephone interviews, where participants were required to recall the falls they had experienced over a particular time frame [18, 23, 27, 28, 31, 33, 34]. Where the time period to be recalled is long, the number of falls reported may be underestimated. Retrospective studies have reported rates of falls per recurrent faller per year of 3.4 and 5.0 [41, 42]. This is similar to the lowest number of falls per recurrent faller per year (4.7) reported by a prospective study included in the present review [23], which collected falls data using a 6 monthly telephone call. In research involving the general older population it has been suggested that notification of falls should occur at least monthly [43]. However, the high prevalence of cognitive impairment [44, 45] and the high frequency of falls experienced by some individuals with PD suggest that a recording system where falls are documented immediately should be used in this population.

Variations in classifying fallers were attributable to differences in the definition of what constitutes a fall as well as differences in the way fall categories were defined. Most studies adhered to aspects of the definition recommended by the Kellogg International Work Group [37] for use with the older population. However, some studies deviated from this definition or did not stipulate how a fall was defined (Table 1). Additionally, this paper found substantial variability in the way that fallers were categorized. For example, nonfallers and single fallers have been combined under the categories of “nonrecurrent fallers” [33, 34] and “nonfallers” [28, 46–49]. While authors use different categories depending on the purpose of their study, the inconsistent categorization of participants is ambiguous and makes comparisons between studies more difficult. This problem could be addressed by standardizing the categories used in future studies. For example, Thomas et al. [17] categorized recurrent fallers according to the number of falls in three months including; “infrequent fallers” (2 to 4 falls), “frequent fallers” (5 to 15 falls), and “very frequent fallers” (>15 falls). The categories of “nonfallers” (0 falls) and single fallers (1 fall) could be added to this to cover the spectrum of fall rates seen in people with PD. 

Substantial variability is also seen in the length of time over which falls data is collected, with the reporting period in the included studies varying from 1 to 29 months. In the present paper, fall rates were adjusted to an approximate yearly rate to facilitate comparison between studies (Table 1). However, this adjustment does not account for disease progression. It seems likely that, as disease severity increases over time, falling rates will also increase [2, 15, 23, 33, 34] until the individual becomes immobile [19]. Consequently, the adjustments used to provide annual fall rates for this review potentially underestimate the rate in studies with a reporting period of less than twelve months [2, 26, 29, 30, 36] and overestimate the rate for the study with a reporting period of longer than twelve months [34]. In order to facilitate comparison of future studies with varied reporting periods, it is recommended that fall data be reported at predetermined intervals. A consensus meeting of experts regarding the general older population recommended that falls be monitored for 12 months [43]. No such review has been undertaken regarding the PD population specifically, although a shorter time period is considered acceptable as people with PD fall more frequently than the general older population [18]. 

This paper has summarized factors associated more strongly with recurrent fallers than single and nonfallers (Table 2). Disease severity was found to be significantly associated with recurrent falls [2, 15, 23, 33, 34] and to be a predictor of future recurrent fallers [2, 34]. A previous review of prospective studies of falling in PD [12] also found that, as the UPDRS motor score increased, the risk of falling increased until the UPDRS score reached around 50 points. Thereafter the risk of falling largely stabilized, with a possible slight reduction in risk with severe disease. The authors speculated that the inclusion of more participants from institutionalized care could result in a further decrease in fall risk with severe disease severity due to the limited mobility of these types of participants. Similarly, the participants included in this paper were mostly community dwelling with mild-to-moderate levels of disease severity. Only one of the included studies [32] examined falling in participants in institutional care. The relationship between disease severity and falls in people with more severe disease, including those requiring care in an institution, requires further investigation. 

Allcock et al. [15] demonstrated an association between fall frequency and impaired attention. It was suggested that impaired attention may contribute to falls by increasing difficulty with performance of concurrent tasks, which may inhibit the performance of compensatory movements to prevent a fall [15]. However, a recent prospective study with a large sample of people with PD (*n* = 263) has found that deterioration in gait under dual task conditions was not associated with future falls [50]. Further research is needed to clarify the clinical implications of the association between cognitive impairment and recurrent falls.

Increased fear of falling has been associated with recurrent falls [18, 33]. This may occur as fear of falling can lead to self-induced restriction of activity [51] resulting in deconditioning and reductions in muscle strength which may increase fall risk [13, 16]. However, there is some evidence that not all recurrent fallers are fearful of falling. In a recent retrospective study [17] two participants who fell very frequently (falling 210 and 360 times each within 3 months) were found to have the lowest fear of falling, even when compared to those who fell rarely (0-1 fall). It was suggested that the experience of very frequent falling with no significant injury or negative consequences could lead to complacency and a resultant lack of fear of falling. Alternatively, low fear of falling could result in risk taking behavior and so contribute to increased incidence of falls. Future prospective studies could seek to clarify this relationship between fear of falling and fall frequency.

This paper identified several factors that have been found to be associated with prospectively recorded recurrent falls, including a positive fall history [2, 15, 18, 23, 33, 34], increased disease severity [2, 15, 33, 34], motor impairment [18, 33] and duration [34], treatment with dopamine agonists [15], increased levodopa dosage [34], cognitive impairment [15, 23], fear of falling [18, 33], freezing of gait [23], impaired mobility [18, 28, 34], and reduced physical activity [34]. While these factors are also known to be associated generally with falls in PD [12, 14, 16, 30], the results of the studies included in this paper suggest that as these factors progress there is an increased tendency for recurrent falls to occur. However, the presence of these associations does not explain why a person with PD who falls occasionally begins to fall recurrently. There is a need for further prospective studies to be conducted which use multivariable regression to investigate the factors that were identified to be relevant in the present paper and their contribution to recurrent falling. Such work would aid in developing an understanding of the causes of recurrent falls. In addition, consideration of factors associated with recurrent falling reported in retrospective studies, including lower limb muscle power [52], impaired motor planning [14, 53], and urinary urge incontinence [42], requires prospective investigation to confirm these relationships. Similarly, the role of medication-related side effects, such as dyskinesia [2, 14] and orthostatic hypotension [54], requires further prospective evaluation regarding their role in recurrent falls in PD.

### 4.1. Clinical Implications

Several risk factors for falls have been found to be more strongly associated with recurrent falls than single falls, suggesting that individuals who fall recurrently may benefit from different fall reduction interventions than single or nonfallers. Some of the factors associated with recurrent falls are potentially modifiable, including cognitive impairment [55, 56], freezing of gait [57], fear of falling [29], reduced mobility [58], reduced physical activity [29, 59], and balance impairment [58]. However, while there is evidence that these factors can be improved with intervention, it remains to be determined whether such improvements would result in reductions in fall frequency, particularly in recurrent fallers.

Given the inconsistent relationship between fear of falling and recurrent falls, it is recommended that fear of falling be assessed in all recurrent fallers and interventions provided accordingly. For example, where fear of falling is found to be high compared to actual fall risk, intervention to reduce fear of falling may be considered. Cognitive behavioral therapy used in conjunction with physical training has been shown to be effective in decreasing fear of falling in the general older population [60] but has not been investigated in the PD population.

## 5. Conclusion

Around 70% of people with PD who fall do so recurrently and many fall very frequently. Recurrent fallers reported 4.7 to 67.6 falls per recurrent faller per year confirming that recurrent falling is a substantial problem for this group. The high variability in the rates of recurrent falls seen in the literature may be attributable to variations in the inclusion criteria used, the method of recording falls, and the way that recurrent fallers are classified including variability in the reporting period used. The large number of falls experienced by some individuals suggests that recurrent fallers as a group should be subdivided into smaller groups based on falls frequency. Further research is needed to ascertain why some recurrent fallers fall much more frequently than others and to investigate falls reduction strategies specific to people with PD who fall recurrently.

## Figures and Tables

**Table 1:** Studies reporting falls prospectively in people with Parkinson's disease.

First author year	Participant number^a^	Inclusion criteria	Exclusion criteria	Fall definition^b^	Reporting period	Classification of falls	Number (%) of fallers^c^	Number of falls^d^	Falls/faller^e^	Falls/faller/yr^f^	Falls/ participant/ yr^g^
Allan 2009 [20]	40 (40)	PD with dementia, >65 years old	Comorbidities affecting ambulation, MMSE ≤ 8, significant visual impairment	2, with or without LOC	12 months	Fall Y/N	36 (90%) ≥ 1 fall	NR	NR	NR	19

Allcock 2009 [15]	164 (176)	Living in the community	NR	2, with or without LOC	12 months	Fall Y/N >1 fall	103 (63%) ≥ 1 fall 32 (20%) = 1 fall **71 (43%) > 1 fall**	736 32 704*	7.1* 1.0* 9.9*	7.1* 1.0* 9.9*	4.5* 0.2* 4.3*

Ashburn 2007^‡^ and Ashburn 2008^‡^ [21, 22]	126 (142)	Independently mobile, living in the community, >1 fall in the last 12 months	Gross cognitive impairment, pain affecting participation, acute medical condition requiring treatment	1, 2, 3	6 months	Fall Y/N >1 fall	95 (75%) ≥ 1 fall 18 (14%) = 1 fall **77 (61%) > 1 fall**	639 18 **621**	6.7 1.0 **8.1**	13.5 2.0 **16.1**	10.3 0.3* **10.0**

Bloem 2001 [2]	59 (61)	Independently mobile, living in the community, clear response to medication, adequate cognition (MMSE ≥ 24)	Comorbidities affecting balance	1, 2	6 months	Fall Y/N >1 fall	30 (51%) ≥ 1 fall 15 (25%) = 1 fall **15 (25%) > 1 fall**	205 15* 190*	6.8* 1.0* 12.7*	13.7* 2.0* 25.3*	6.9* 0.5* 6.4*

Camicioli 2010 [23]	52 (52)	≥65 years old	Dementia, unstable medical illness, other illness affecting thinking or memory	NR	12 months	Fall Y/N >1 fall	21 (40%) ≥ 1 fall 6 (12%) = 1 fall **15 (29%) > 1 fall**	76* 6 70*	3.6* 1.0* 4.7*	3.6* 1.0* 4.7*	1.5* 0.1* 1.3*
Chung 2010^‡^ [24]	19 (23)	Responsive to levodopa, ≥2 falls or near falls per week, walk independently indoors with or without an aid	Freezing, non-CNS contributors to falls, using cholinesterase inhibitors/ anticholinergic drugs or sedatives, MMSE < 25, any unstable medical condition	1, 2, 3	6 weeks Donezepil 6 weeks placebo	NR	NR NR	104* 199*	NR NR	NR NR	47.3* 91*

Cole 2010 [25]	49 (49)	Nil	Recent/recurrent injury or surgery, unable to ambulate independently with/without a walking aid, significant visual or cognitive impairment (MMSE < 24)	1, 2, 3	12 months	Fall Y/N >1 fall	32 (65%) ≥1 fall 11 (22%) = 1 fall **21(43%) > 1 fall**	NR NR NR	NR NR NR	NR NR NR	NR NR NR

Donovan 2011^‡^ [26]	23 (32)	Independently mobile but requiring a walking aid, experience FOG	Syncopal episode in prior 6 months, prior exposure to laserlight visual cueing device	1, 2	1 to 2 months baseline 1 month after baseline	Fall Y/N	10 (43%) ≥ 1 fall 10 (43%) ≥ 1 fall	NR	NR	168* 110.2*	73* 47.9*

Fink 2005 [27]	49 (52) men only	≥65 years old, living in the community	Unable to walk independently, bilateral hip replacement	NR	12 months	>1 fall	**14 (29%) > 1 fall**	NR	NR	NR	NR
Foreman 2011 [28]	36 (36)	>40 years old, independently mobile, gait hypokinesia present, sufficient cognition (MMSE > 23), taking Carbidopa or Levodopa	Had surgical management of PD, uncontrolled motor fluctuations, comorbidities affecting mobility or balance	Person comes to rest on ground	≥6 months	0 or 1 fall >1 fall	**22 (61%) > 1 fall**	NR	NR	NR	NR

Goodwin 2011^‡^ [29]	122 (130) baseline 125 (130) post-baseline	>1 fall in previous year, walk independently indoors with or without a walking aid	Comorbidities affecting ability to exercise safely, unable to follow written or verbal instructions in English	1, 2	10 weeks baseline 20 weeks after baseline	Fall Y/N	109 (84%) ≥ 1 fall 107 (86%) ≥ 1 fall	3453* 5488*	31.7* 51.3*	164.7* 133.4*	138.1* 114.2*

Gray 2000^†^ [30]	118 (118)	Able to stand and walk a short distance with or without a walking aid	Comorbidities that could predispose to falls, cognitive or writing deficit unless caregiver able to assist completion of falls diary	included “near falls” as a fall	12 weeks	Fall Y/N 1 fall 2 to 3 falls 4 to 5 falls >5 falls	Unclear as near falls included	144	NR	NR	5.3*

Hayashi 2010 [31]	20 (20)	Hoehn and Yahr Stage ≥ 2, responsive to levodopa, receiving regular outpatient treatment every month	Other neurological disease, significant dementia, or autonomic dysfunction	NR	12 months	Fall Y/N >1 fall >5 falls	**13 (65%) > 1 fall** **6 (30%) > 5 falls**	NR NR	NR NR	NR NR	NR NR
Kerr 2010 [16]	101 (106)	Walking independently without aid, living in the community independently	Nil	1, 2, 3	6 months	Fall Y/N >1 fall	48 (48%) ≥ 1 fall 24 (24%) = 1 fall **24 (24%) > 1 fall**	NR NR NR	NR NR NR	NR NR NR	NR NR NR

Latt 2009 [13]	113 (113)	Living in the community	Unable to walk without aid, atypical Parkinsonism, insufficient cognition (MMSE < 24)	1, 2	12 months	Fall Y/N	51 (45%) ≥ 1 fall	2160	42.4*	42.4*	19.1*

Lord 2003 [32]	57 (57)	Living in residential elderly care facility	Bedbound	1, 2, 3	6–29 months Followed until death or for at least 6 months Mean = 15.3 ± 7.5 months	Fall Y/N 1 fall 2 to 4 falls 5 falls	36 (63%) ≥ 1 fall 6 (11%) = 1 fall **30 (53%) **>** 1 fall**	NR for PD group alone 6 NR for PD group alone	NR 1.0 NR	NR NR NR	NR NR NR

Mak 2009 [33]	70 (72)	Living in the community, 40–85 years old, medically stable, walk 3 × 6 m with or without a walking aid	Other neurological conditions, communication deficit, impaired cognition (MMSE < 24), postural hypotension, visual disturbance, vestibular dysfunction, other comorbidities limiting locomotion or balance	2, 3	12 months	0 or 1 fall >1 fall	**15 (21%) **>** 1 fall**	NR	NR	NR	NR
Mak 2010 [18]	72 (74)	Age ≥ 40 yrs, medically stable, walking independently with or without a walking aid	Other neurological conditions, communication deficit, insufficient cognition (MMSE < 24), visual disturbance, vestibular dysfunction, comorbidities limiting locomotion or balance	2, 3	12 months	1 fall >1 fall	25 (35%) ≥ 1 fall 12 (17%) = 1 fall **13 (18%) > 1 fall**	133 12 121*	5.3* 1.0* 9.3*	5.3* 1.0* 9.3*	1.8* 0.2* 1.7*

Matinolli 2011 [34]	125 (125)	Able to stand unsupported	Placed in long-term institutional care	1, 2	24 months	Fall Y/N 1 fall 2–5 falls 6–10 falls 11–100 falls >208 falls	79 (63%) ≥ 1 fall 20 (16%) = 1 fall **59 (47%) > 1 fall**	3125 20 3105*	39.6* 1.0* 52.6*	19.8* 0.5* 26.3*	12.5* 0.1* 12.4*

Nilsson 2011^‡^ [35]	19 (20)	Idiopathic PD selected for bilateral DBS of the subthalamic nuclei, responsive to levodopa but with insufficient effect, normal brain MRI	Signs of dementia or severe cognitive decline, severe comorbidity, electrode replacement required within 6 months of surgery	1, 2	12 weeks prior to Sx 12 months after Sx	Fall Y/N >1 fall	10 (53%) ≥ 1 fall 5 (26%) = 1 fall **5 (26%) > 1 fall** 14 (74%) ≥ 1 fall 2 (11%) = 1 fall **12 (63%) > 1 fal**l	83 5 **78** 204 2 202*	8.3* 1.0 15.6* 14.6* 2.0* 16.8*	36.0* 4.3* 67.6* 14.6* 2.0* 16.8*	18.9* 1.1* 17.8* 10.7* 0.1* 10.6*

Smania 2010^‡^ [36]	55 (64)	Hoehn and Yahr Stage 3 or 4, able to rise from chair or bed independently, MMSE > 23	Other neurological conditions or conditions that could interfere with the study	1, 2, 3	1 month Baseline 1 month post 1 month follow-up	NR	NR NR NR	1415* 1329* 1337*	NR NR NR	NR NR NR	308.7* 290.0* 291.7*

Wood 2002^‡^ [10]	101 (109)	Living in the community	Bedbound, severe medical instability	1, 2, 3	12 months	Fall Y/N >1 fall	69 (68%) ≥ 1 fall 18 (18%) = 1 fall **51 (50%) > 1 fall**	585 18 567*	8.5* 1.0* 11.1*	8.5* 1.0* 11.1*	5.8* 0.2* 5.6*

^a^Participant number—number reported (number recruited).

^b^Fall definition [37]—1 = unintentional/unexpected change in position, 2 = person comes to rest on lower level, 3 = not as a result of a major intrinsic event or overwhelming hazard.

^c^Number (%) of fallers—reported for fallers (single + recurrent), single fallers, and recurrent fallers.

^d^Number of falls—recurrent falls are shown in bold.

^e^Falls/faller—number of falls divided by the number of fallers, calculated for each reported fall category.

^f^Falls/faller/yr—number of falls divided by the number of fallers, adjusted to give an approximate yearly rate.

^g^Falls/participant/yr—number of falls divided by the number of participants in the study, calculated for each reported fall category, and adjusted to give an approximate yearly rate.

Bold font indicates data pertaining to recurrent falls; ^‡^study is an intervention trial; *data calculated from published paper; ^†^definition of falls included all of the following: “near falls” (i.e., fall initiated but arrested by support from a wall, railing, other person, etc.), “whole body falls,” falls to the hand or knee, and falls that were unable to be categorized based on the information reported by the participant. Only data for “whole body falls” and falls to the hand or knee are reported in this table.

PD: Parkinson's disease; LOC: loss of consciousness; NR: not reported or insufficient detail to calculate; Y: yes; N: no; FOG: freezing of gait; Sx: surgery.

**Table 2:** Factors associated with recurrent falls in Parkinson's disease.

First author year study aim	Participant number^a^ Tested ON or OFF	Disease severity	Reporting period	Classification of participants	Number per falls classification	Variables examined	Analyses	Results
Allcock et al. 2009 [15] Determine whether measures of attention were associated with falls	164 (176) OFF	Unclear	12 months	0 falls 1 fall >1 fall	61 (37%) 32 (20%) 71 (43%)	Cognitive impairment Demographics Disease severity Fall history Nonmotor impairments PD medications Other medications	Negative binomial regression	Significant explanatory variables explaining fall frequency (i) Disease severity (UPDRS) (ii) Dopamine agonists (iii) Cognitive impairment (a) Power of attention (b) Cognitive reaction time (c) Reaction time variability (iv) Fall history

Bloem et al. 2001 [2] Identify risk factors associated with falls and prediction of falls, particularly in relation to balance and gait	59 (61) ON	Mild-moderately severe	6 months	0-1 fall >1 fall	44 (75%) 15 (25%)	Activities of daily living Demographics Disease duration Disease severity Fall history Fear of falling Medications Mobility and use of aids Motor impairments Multiple task performance	Stepwise forward logistic regression	Recurrent fallers best predicted by the following (i) Disease severity (H&Y) (ii) Fall history

Camicioli and Majumdar 2010 [23] Identify risk factors associated with falls, with a focus on cognitive impairment	52 (52) ON	Mild-moderate	12 months	≥1 fall >1 fall	21 (40%) 15 (29%)	Cognitive impairment Demographics Disease severity Fall history Fear of falling Gait parameters Motor impairments Nonmotor impairments PD medications	Univariate analysis	Factors associated with an increased risk of recurrent falls (i) Cognitive impairment (CCDRSum) (ii) Fall history (iii) Disease severity (H&Y) (iv) Freezing (UPDRS item)

Foreman et al. 2011 [28] Examine the Functional Gait Assessment, the pull test, and the timed up and go and their relation to falls	36 (36) OFF and ON	Mild-moderately severe	≥6 months	0-1 fall >1 fall	14 (39%) 22 (61%)	Demographics Disease duration Disease severity Mobility Motor impairments	Receiver operating characteristic curve	Interpretation of performance when OFF provided more accurate prediction of fall status than the ON condition
							Between-group comparisons	Compared to single + nonfallers, recurrent fallers had the following (i) Worse Functional Gait Assessment scores when ON and when OFF (ii) Slower timed up and go when OFF
Mak and Pang 2009 [33] Examine whether fear of falling could independently predict recurrent falls	70 (72) ON	Moderate	12 months	0-1 fall >1 fall	55 (79%) 15 (21%)	Demographics Disease duration Disease severity Fall history Fear of falling Medications Mobility Nonmotor impairments	Stepwise discriminant analysis	For predicting future recurrent fallers (i) Fall history strongest predictor (ii) UPDRS motor score and fear of falling (ABC) remain significant after adjusting for fall history
							Receiver operating characteristic curve	For identifying recurrent fallers (i) ABC cut-off score of 69 (sensitivity 93%, specificity 67%) and UPDRS motor score of 32 (sensitivity 47%, specificity 94%) provide the best combination
							Between-group comparisons	Compared to single + nonfallers, recurrent fallers had the following (i) Increased disease severity (H&Y) (ii) Higher UPDRS motor scores (iii) Increased fear of falling

Mak and Pang 2010 [18] Compare fall characteristics between single and recurrent fallers	72 (74) ON	Mild-moderate	12 months	0 falls 1 fall >1 fall	47 (65%) 12 (17%) 13 (18%)	21 variables including Anthropometrics Demographics Disease duration Disease severity Fall history Fear of falling Habitual physical activity Mobility and use of aids Motor impairments Nonmotor impairments PD medications	Between-group comparisons	Compared to single fallers, recurrent fallers had the following (i) More previous falls (ii) Increased PD motor impairments (UPDRS) (iii) Reduced walking capacity (6 MWD) (iv) Increased use of walking aids (v) Reduced speed of sit-to-stand (vi) Increased fear of falling (ABC) (vii) A higher proportion of falls occurring indoors at home as opposed to outdoors

Matinolli et al. 2011 [34] Identify balance and mobility related risk factors for recurrent falling	125 (125) ON	Mild-moderate	24 months	0-1 fall >1 fall 0 falls 1 fall 2–5 falls 6–10 falls 11–100 falls >208 falls	66 (53%) 59 (47%) 46 (37%) 20 (16%) 22 (17%) 16 (13%) 15 (12%) 6 (5%)	Comorbidities Cognitive impairment Demographics Disease severity Fall history Fear of falling Habitual physical activity Mobility and use of aids Motor impairments Nonmotor impairments Other medications PD medications	Forward stepwise regression	Significant risk factors in the final multivariable model predicting recurrent falls (i) Fall history (ii) Disease severity (UPDRS II)
							Between-group comparisons	Compared to single + nonfallers, recurrent fallers had the following (i) Longer disease duration (ii) Increased disease severity (H&Y and UPDRS ADL score, motor score and total) (iii) Presence of freezing of gait (iv) More falls unrelated to freezing of gait (UPDRS item 13) (v) Experienced recent falls (vi) Higher levodopa dose (vii) Decreased physical activity (viii) Reduced mobility (slowed walking speed and TUG) (ix) Increased use of walking aids (x) Increased postural sway

^a^Participant number-number reported (number recruited).

NR: not reported; UPDRS: Unified Parkinson's Disease Rating Scale; H&Y: Hoehn and Yahr stage; CCDRSum: Caregiver-rated Clinical Dementia Rating Scale; ABC: Activities-Specific Balance Confidence Scale; 6MWD: 6-minute walk distance; ADL: activities of daily living; TUG: timed up and go.
